# A Case Study of a Rapidly Progressive Cellulitis of the Penis and Scrotum in a Two-Month-Old Infant

**DOI:** 10.7759/cureus.32255

**Published:** 2022-12-06

**Authors:** Michael A Larsen, Alexander H Hogan, Hassan El Chebib

**Affiliations:** 1 Pediatrics, Frank H. Netter MD School of Medicine, North Haven, USA; 2 Pediatrics, Division of Hospital Medicine, Connecticut Children’s Medical Center, Hartford, USA; 3 Pediatrics, School of Medicine, University of Connecticut, Farmington, USA; 4 Infectious Diseases and Immunology, Connecticut Children’s Medical Center, Hartford, USA; 5 Infectious Diseases, School of Medicine, University of Connecticut, Farmington, USA; 6 Infectious Diseases, Pediatric Infectious Diseases Fellowship Program, University of Connecticut, Hartford, USA

**Keywords:** penis and scrotum, rapidly progressive cellulitis, case study, penis, scrotum, infant, cellulitis

## Abstract

A two-month-old infant presented with rapidly progressive cellulitis of the penis and scrotum without a history of trauma, circumcision, or previous infection. After multiple failed antibiotic regimens covering common pathogens associated with cellulitis, a combination of ceftazidime and clindamycin was used to treat his infection. The previous evidence of anaerobic bacteria being implicated in male genitourinary infections and the antibiotic course necessary in this patient’s treatment indicate that infantile scrotal cellulitis could require a distinct approach from typical skin and soft tissue infections.

## Introduction

Cellulitis is a rare cause of pediatric acute scrotal pain, causing less than 0.5% of cases [1]. All prior case reports documented preceding trauma, including circumcision, as a potential nidus of infection [2-4]. Typically, skin and soft tissue infections (SSTIs) are caused by *Staphylococcus aureus* and group A *Streptococcus *(GAS) [5]; however, scrotal cellulitis has additionally been associated with gastrointestinal bacteria and group B *Streptococcus* (GBS) [1-3,6,7]. The fact that anaerobes are implicated in genital infections is unsurprising, given the close proximity of the gastrointestinal tract, which harbors bacteria that are predominantly obligate anaerobes [6]. Further, anaerobes, predominantly *Bacteroides *sp.*, *are found in the cultures taken from roughly 85% of infected areas throughout the body and from 88% of suppurative infections of the male genitourinary tract [4,6].

We report a two-month-old infant with rapidly progressing scrotal cellulitis despite antibiotic coverage for typical SSTIs and the absence of any recent trauma. The patient we present illustrates the need to consider broader antibiotic coverage in this high-risk area of the body.

## Case presentation

At two months of age, this uncircumcised boy, whose past medical history was significant for chordee and bilateral hip dysplasia, being treated with a Pavlik harness, developed swelling of his left scrotum. His mother brought him to the emergency department (ED) and he was found to be afebrile, and overall well-appearing, apart from significant scrotal swelling. An ultrasound (US) of the scrotum revealed a left-sided hydrocele without evidence of testicular torsion. He was sent home with plans to follow up with his pediatrician in five days. On the morning of the follow-up visit, his mother noted increased edema and erythema of the scrotum and penis and a new fever of 101.5°F (38.6°C) rectally. These symptoms worsened dramatically over the course of the morning. The patient was seen by his pediatrician and immediately referred back to the ED, where his Pavlik harness was removed and not used again throughout his hospital course.

**Figure 1:**
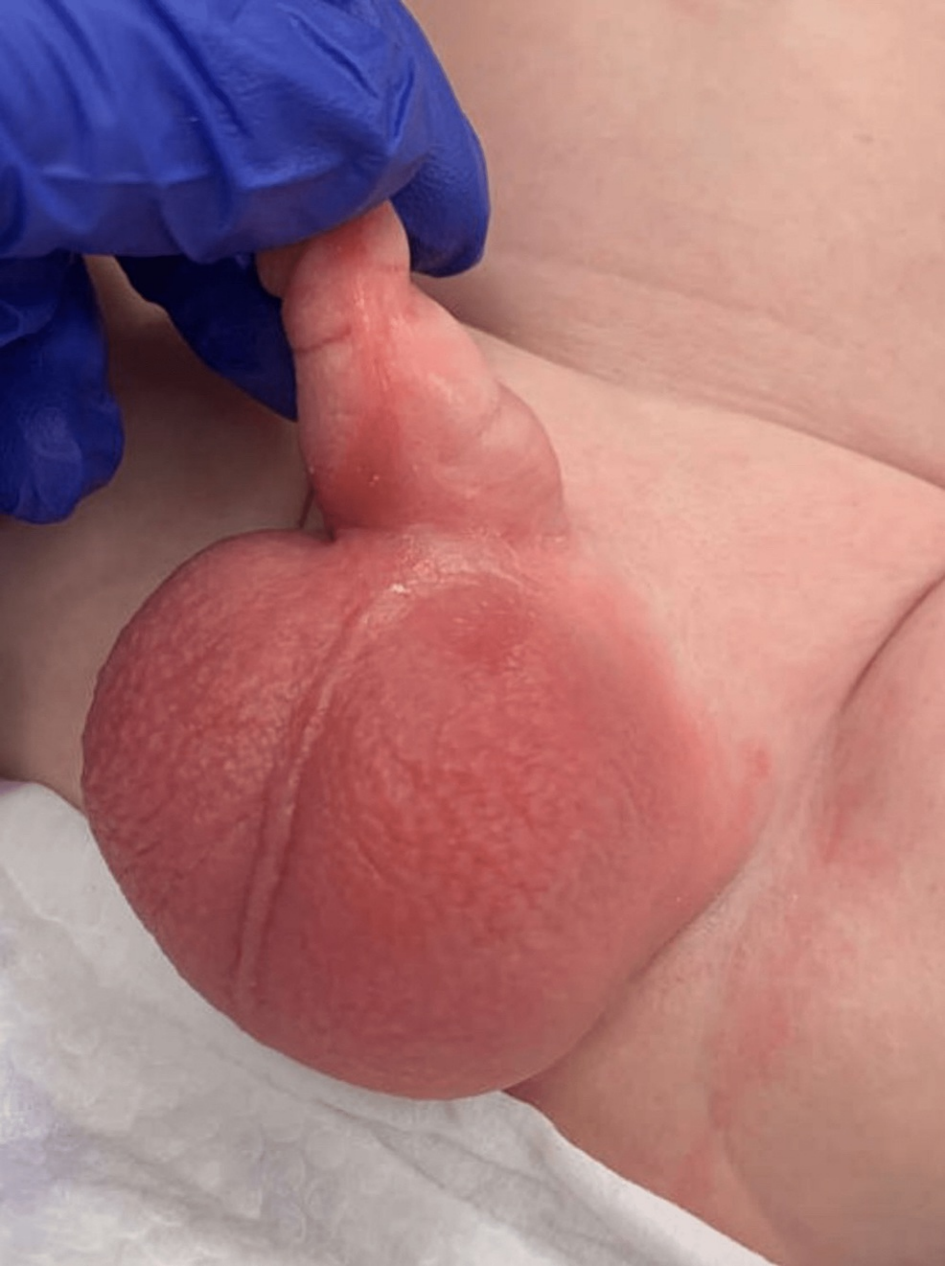
Initial presentation of scrotal cellulitis in an infant The scrotum and phallus show localized and prominent edema and erythema without exudate.

In the ED, the patient was irritable and crying, but not toxic-appearing. His vitals were as follows: fever of 100.8°F (38.2°C), blood pressure of 82/59 mmHg, pulse rate of 67 bpm, a respiration rate of 30 breaths per minute, and oxygen saturation of 100% on room air. His physical examination, apart from his genital exam, did not reveal any abnormalities. A physical exam of his genitals revealed an edematous and erythematous scrotum with rugae and thickened scrotal skin (Figure 1). Both his phallus and scrotum were exquisitely tender to palpation during the examination. The edema and erythema had fine borders and did not extend beyond his genitals. His left testicle was more edematous than his right. No crepitus or skin breakdown was noted. His penis was also erythematous and swollen, mostly in the proximal segment. His known left hydrocele was evident through transillumination. His glans was unable to be expressed due to phimosis and chordee, but no discharge was noted.

Doppler ultrasound of his penis and scrotum was performed emergently to assess the possibility of testicular torsion. It revealed no evidence of torsion but reaffirmed the presence of his known left-sided hydrocele. It was also remarkable for bilateral scrotal soft tissue swelling more notably on the right side.

The patient’s complete blood count (CBC) at this time is represented in Table 1 and revealed normocytic anemia with a low mean platelet volume. The basic metabolic panel (BMP) revealed an elevated anion gap metabolic acidosis with simultaneous pre-renal azotemia, which is represented in Table 2. The patient's C-reactive protein (CRP) at this time was 3.80 mg/dL (reference range: 0.00-0.49 mg/dL). Erythrocyte sedimentation rate and lactate were not obtained.

**Table 1:** Complete blood count values upon admission to inpatient pediatrics of the patient showing normocytic anemia with low mean platelet volume (-) = below the reference range.

Component	Reference range & units	Patient's value
WBC	5.0-17.0 Thou/uL	8.2
Platelets	150-400 Thou/uL	387
Hemoglobin	11.0-16.3 g/dL	10.1 (-)
Hematocrit	31.5-48.9 %	29.7 (-)
RBC	3.50-5.30 Mil/uL	3.28 (-)
Mean corpuscular volume	92-110 fL	91 (-)
Mean corpuscular hemoglobin	25.0-35.0 pg	30.8
Mean corpuscular hemoglobin concentration	30.0-36.0 g/dL	34
Red cell distribution width	11.5-14.5 %	12.7
Mean platelet volume	9.4-12.5 fL	8.9 (-)
Neutrophils	%	67.1
Immature granulocyte	%	0.5
Lymphocyte	%	19.8
Monocyte	%	12.6
Eosinophil	%	0
Basophil	%	0

**Table 2:** Basic metabolic panel at admission revealing pre-renal azotemia and an elevated anion gap metabolic acidosis (-) = lab value is below the reference range; (+) = lab value is above the reference range. * This was not a fasting glucose and is thus not accurately characterized by the reference range.

Component	Reference range & units	Patient's values
Glucose	65-99 mg/dL	118*
Blood urea nitrogen (BUN)	5-18 mg/dL	12
Creatinine	0.2-0.7 mg/dL	0.2
Sodium	136-145 mmol/L	139
Potassium	4.1-5.3 mmol/L	5
Chloride	98-106 mmol/L	102
CO2	20-28 mmol/L	18 (-)
Anion gap	7-17	19 (+)
Calcium	7.6-10.4 mg/dL	10.2
BUN/creatinine ratio	10.0-25.0 ratio	60 (+)

Pediatric urology was consulted in the ED and recommended the patient be admitted for IV antibiotics and monitoring given the rapid progression and systemic nature of his symptoms. The patient’s nutrition was maintained on the mother’s milk or formula. The patient’s fever was managed with acetaminophen oral suspension every six hours as needed.

The initial antibiotic regimen was with IV cefazolin 100 mg/kg/day every eight hours for coverage of GAS and *S. aureus* [5]. Unfortunately, the cellulitis worsened on this regimen throughout the day. The erythema and edema continued to increase, and within 12 hours of admission, the patient’s CRP had risen to 6.87 mg/L (reference range: 0.00-0.49 mg/dL).

Given the worsening of the patient’s symptoms, the pediatric infectious disease team was consulted that recommended IV ceftriaxone at 75 mg/kg/day every 12 hours to provide broader coverage of urinary and gastrointestinal pathogens. Standard blood cultures were also obtained due to the rapid progression of his edema and the presence of systemic symptoms (fever). No anaerobic culture was taken at this time due to low initial suspicion of abnormal pathogens for skin infection. His blood culture remained sterile, though these cultures were taken after the patient’s first dose of ceftriaxone. Urinalysis and urine culture were obtained via bag due to the degree of penile swelling. Urinalysis was normal and urine culture remained sterile.

Despite this therapy, the patient’s symptoms continued to worsen overnight and had progressed to involve his perineum and bilateral gluteal cheeks by day two of admission. Consequently, the patient was transitioned to a regimen of IV ceftazidime 150 mg/kg/day and IV clindamycin 10 mg/kg/day to attain better gram-positive, anaerobic, and methicillin-resistant *S. aureus *(MRSA) coverage. This regimen was continued for three days with a noted improvement in the patient’s symptoms. The scrotum became markedly less edematous with a decrease in erythema throughout the region; the patient also became more active, was more willing to eat, and had less tenderness to palpation during the examination. To note, clindamycin was selected over metronidazole for anaerobic coverage given the shortage of IV metronidazole at the time necessitating strict use of IV metronidazole in selected clinical conditions (for example, complicated sinusitis with intra-cranial abscess). The antibiotic course used throughout the hospital stay is summarized in Table 3.

**Table 3:** Progression of antibacterial treatment regimen over the course of the patient's clinical stay Over the course of the patient's stay, the antibacterial regimen was increased to include better anaerobe and methicillin-resistant *Staphylococcus aureus* (MRSA) coverage due to the failure of previous antibiotic regimens.

Hospital day	AM/PM	Treatment change	Rational	Outcome
1	AM	Initiated IV cefazolin 100 mg/kg/day every eight hours	Coverage of *Staphylococcus aureus* and *Streptococcus pyogenes*	Continued patient deterioration and worsening of symptoms
1	PM	Changed cefazolin → IV ceftriaxone 75 mg/kg/day every 12 hours	Continued symptom progression and increasing C-reactive protein despite cefazolin treatment. It was recommended better anaerobe coverage was required due to suspicion that gastrointestinal and/or urinary tract bacteria were involved	Continued patient deterioration and worsening of symptoms
2-4	AM	Changed from ceftriaxone → ceftazidime 150 mg/kg/day and IV clindamycin 10 mg/kg/day	Continued symptom progression despite ceftriaxone therapy. Desire to achieve better gram-positive, anaerobe, and MRSA coverage	Improvement of patient symptoms over the course of three days of treatment
Day 5, discharge	AM	Discontinued IV ceftazidime and IV clindamycin in favor of oral ciprofloxacin and metronidazole for 10 days following discharge	Resolution of symptoms on IV antibiotics and tolerating oral (PO) well. Discharged with PO medications appropriately covering anaerobes and gram-positive organisms	Continued outpatient improvement of symptoms and full return to baseline over the course of treatment

He was discharged on the morning of day five of the clinical course with a continued decrease in erythema, edema, and tenderness to palpation in his genital region and with vitals within normal limits on oral ciprofloxacin and oral metronidazole suspension for a total course of 10 days. His mother was given instructions to follow up with their outpatient provider.

At a virtual check-in with the patient's mother via Zoom (Zoom Video Communications, San Jose, CA) two weeks after the patient’s admission, the patient’s mother reported they followed the recommended outpatient antibiotic course. She states the patient continued improving steadily following discharge and that he has achieved a full recovery to baseline.

The pediatric infectious diseases and pediatric urology teams were consulted throughout the treatment course, and their recommendations were followed. Neither felt the surgical intervention was indicated at any time throughout the patient’s hospital course.

## Discussion

The differential diagnosis of the acute scrotum is broad and requires a multidisciplinary team to consider all potential evaluations and treatments. Testicular and appendix testes torsion were considered and ruled out emergently via Doppler (US). Epididymitis was also considered but was less likely due to the bilateral nature of his symptoms, rapid progression, and systemic symptoms. Genital erysipelas was considered as erythematous and edematous rash had well-defined borders. However, a deeper skin infection was more likely given the thickened scrotal skin. Additionally, GAS, the most common etiology of erysipelas, would have very likely responded to the initial therapy of IV cefazolin [8]. Several cases of Fournier's gangrene have been documented in case reports [7]. Here, the patient showed no signs of crepitus or skin breakdown, making the progression to a gangrenous state less likely. These differentials were considered but deemed much less likely; this, coupled with the rapid improvement on broad-spectrum antibiotics, suggested cellulitis was the most likely final diagnosis in this patient.

Previous instances of scrotal cellulitis were preceded by severe diaper rash or trauma, including circumcision [2,3,7]. These circumstances provide an avenue of penetration and a source of infection and inflammation. This case is different in that these aforementioned circumstances were not present. Furthermore, the patient's rapid progression of erythema, edema, and tenderness without resolution of symptoms given the extensive initial antibiotic courses also suggests a unique case. Though this infection was likely idiopathic, several hypotheses regarding factors that increased his risk are presented here. The patient suffers from chordee without hypospadias and left hydrocele. One could argue hydrocele could introduce a path by which gastrointestinal bacteria could invade the scrotum. Per our review, neither of these conditions is associated with an increased infection rate in the genital area. However, his chordee prevented the patient from being circumcised, which mildly increases his chance of urinary tract and genital infections [9]. The Pavlik harness could have increased the risk of skin breakdown due to rubbing of the straps in the genital area; however, no evidence of skin breakdown was found on the physical exam and the harness was applied on top of his diaper limiting direct contact of the harness with the patient’s skin.

Previous reports have identified GBS and gastrointestinal anaerobes as etiologies of cellulitis in the scrotum that are atypical of cellulitis [2,3,5,7], which when coupled with our patient’s improvement with broad-spectrum antibiotics, suggests the need for broader coverage than typically used for SSTIs. Previously successful regimens included ampicillin, gentamicin, and clindamycin [2]. The successful regimen for this patient included ceftazidime and clindamycin. As such, it appears a regimen with wide gram-positive and good anaerobic coverage could be a beneficial choice in pediatric scrotal cellulitis, particularly in cases of clinical worsening on conventional SSTI coverage.

A notable weakness of this report is the lack of a microbiologic diagnosis, which would have confirmed an anaerobic pathogen as the etiology of this patient's infection. It is likely that the lack of anaerobic analysis in blood cultures contributed to a sterile culture. However, this case contributes further evidence for the need for anaerobic coverage when selecting an antibiotic regimen for scrotal cellulitis on top of the standard therapy for gram-positive bacteria. It should similarly be noted that cellulitis has been known to take over 24 hours to show signs of improvement despite appropriate antibiotic therapy [10]. This possibility was discussed with infectious disease; however, it was felt at the time that broadening antibiotic therapy was appropriate given the rapid progression of symptoms despite therapy for classic skin flora.

## Conclusions

Given that this patient worsened on standard cellulitis therapy, traditional approaches to cellulitis may be insufficient for instances of scrotal cellulitis. Our treatment course ultimately necessitated the use of ceftazidime and clindamycin. As such, an initial approach to cellulitis of the genitals in infants could benefit from the inclusion of an antibiotic regimen with wide gram-positive and anaerobic coverage.
